# Functional Relevance for Associations between Genetic Variants and Systemic Lupus Erythematosus

**DOI:** 10.1371/journal.pone.0053037

**Published:** 2013-01-14

**Authors:** Fei-Yan Deng, Shu-Feng Lei, Yong-Hong Zhang, Zeng-Li Zhang, Yu-Fan Guo

**Affiliations:** 1 Center for Genetic Epidemiology and Genomics, Soochow University, Suzhou, Jiangsu, P. R. China; 2 Department of Epidemiology, School of Public Health, Soochow University, Suzhou, Jiangsu, P. R. China; 3 Department of Rheumatology, the First Affiliated Hospital of Soochow University, Suzhou, Jiangsu, P. R. China; University of Michigan Medical School, United States of America

## Abstract

Systemic lupus erythematosus (SLE) is a serious prototype autoimmune disease characterized by chronic inflammation, auto-antibody production and multi-organ damage. Recent association studies have identified a long list of loci that were associated with SLE with relatively high statistical power. However, most of them only established the statistical associations of genetic markers and SLE at the DNA level without supporting evidence of functional relevance. Here, using publically available datasets, we performed integrative analyses (gene relationship across implicated loci analysis, differential gene expression analysis and functional annotation clustering analysis) and combined with expression quantitative trait loci (eQTLs) results to dissect functional mechanisms underlying the associations for SLE. We found that 14 SNPs, which were significantly associated with SLE in previous studies, have cis-regulation effects on four eQTL genes (HLA-DQA1, HLA-DQB1, HLA-DQB2, and IRF5) that were also differentially expressed in SLE-related cell groups. The functional evidence, taken together, suggested the functional mechanisms underlying the associations of 14 SNPs and SLE. The study may serve as an example of mining publically available datasets and results in validation of significant disease-association results. Utilization of public data resources for integrative analyses may provide novel insights into the molecular genetic mechanisms underlying human diseases.

## Introduction

Systemic lupus erythematosus (SLE) is a serious prototype autoimmune disease characterized by chronic inflammation, auto-antibody production and multi-organ damage. The estimated incidence of SLE ranges from 2.0–7.6 cases per 100,000 persons per year [1]. The overall prevalence of SLE in the United States ranges from 14.6–50 cases per 100,000 persons [1]. Heritable components play an important role in pathogenesis of SLE, as evidenced by up to 30 times higher risk for siblings of affected individuals than that for the general population and further increased risk for monozygotic twins [2]–[5].

Dissecting the genetic basis of SLE remains one of great challenges in human genetics, because of complex nature of genetic determination, including polygenic determinations, gene by gene and gene by environment interactions. Recently, there has been great progress in identification of genetic susceptibility in SLE [6]–[13]. A great number of genetic loci associated with SLE were identified with relatively high statistical power. However, most of these studies only established statistical associations of genetic markers and SLE at the DNA level without exploring functional mechanisms underlying the associations. Such established associations usually do not provide immediate insights into functions of genes or regulation of gene expression that bridges gene code information and SLE phenotype directly.

Currently, numerous public gene expression datasets (e.g., www.ncbi.nlm.nih.gov/geo) are available, which were used to identify genes relevant to SLE in the original studies [14]. These datasets will serve as important resources for further data mining to provide supplementary functional evidence to bridge the functional link between genetic markers and SLE.

Based on the publically available datasets and results, this study aims to dissect functional mechanisms underlying the associations identified in previous studies by performing integrative analyses (gene relationships among implicated loci (GRAIL), differential gene expression analysis and functional annotation clustering analysis) and by combining the findings with expression quantitative trait loci (eQTLs) analysis results.

## Materials and Methods

### Selection of SLE-Associated SNPs

GWAS Integrator [15] and Phenotype-Genotype Integrator (PheGenI) (www.ncbi.nlm.nih.gov/gap/PheGenI/) are two bioinformatics tools that provide robust lookup and analytic functionalities for published GWAS and meta-analysis studies. We used the phenotype “systemic lupus erythematosus” to search the two databases and found 135 interesting associations for 128 unique SNPs and SLE with p<10^−7^ (**Table S1**) [6]–[13]. Seven SNPs have replicate associations in independent studies.

### Gene Relationship across Implicated Loci (GRAIL)

In most situations, confidently significant SNP means at least one susceptible gene or functional locus is located within the nearby region of the SNP, which often contains multiple genes. A commonly used approach for identification of candidate gene is to select nearby genes physically located at two sides of each associated SNP as candidate genes for further functional studies. To increase chances of finding susceptible genes, we used the GRAIL [16] to prioritize and identify susceptible candidate genes near SLE-associated SNPs. This approach automatically assesses the degree of functional connectivity with the other candidate genes that were chosen for the analysis using 250,000 PubMed abstracts, then prioritizes and identifies the best candidate gene around associated SNP.

### eQTL Analysis

As is commonly recognized, variants at the DNA level may lead to variations in quantifiable intermediate phenotypes (such as RNA level), which subsequently result in variations in end-point phenotypes, such as susceptibility to SLE. Several recent large-scale studies have identified thousands of putative eQTLs in multiple tissues or cells including liver, lymphoblastoid cell lines(LCLs), monocytes, T cells, brain and fibroblasts [17]–[23], which were summarized and archived in databases for quick and easy searching and comparison by web browsers (eQTL Browser: http://eqtl.uchicago.edu/cgi-bin/gbrowse/eqtl/; GTEx eQTL Browser: www.ncbi.nlm.nih.gov/gtex/GTEX2/gtex.cgi). To investigate the functional mechanisms underlying the above selected associations, we first searched the two databases for eQTLs to evaluate whether the above detected SNPs influence transcript levels of genes as cis-effect regulators (eSNP) in multiple SLE related cells (monocytes, LCLs and fibroblasts).

### Differential Expression Analysis for SLE-Associated Genes

We performed differential expression analyses for the identified eQTL genes. First, we downloaded three publically available expression data sets from GEO Datasets (www.ncbi.nlm.nih.gov/geo) (GSE numbers: GSE37356, GSE20864). These studies were performed with original purposes of identifying genes underlying SLE using a design of SLE case and control in multiple tissues or cells, e.g., macrophages, mononuclear cells, and peripheral blood cells [14]. The experiment procedures and data analyses including normalization of raw data were detailed in the original publications [14]. By using the normalized data available on the public databases, T-tests were performed to identify differential expression genes (DEGs) by comparing means of the gene expression signals in SLE cases and controls.

### Functional prediction for SNP

For the significant SNPs identified and other interesting SNPs, we utilized the FASTSNP program (http://fastsnp.ibms.sinica.edu.tw) to analyze and predict their functions. FASTSNP provides up-to-date information about known and potential functional effect of SNPs [24].

### Functional annotation clustering analysis

Functional annotation clustering analyses were performed to explore functional similarity of the associated genes and to cluster them in particular biological GO terms and functional pathways as defined by the Gene Ontology (GO) project and KEGG database, respectively. The significantly associated genes were functionally annotated using the Database for Annotation, Visualization and Integrated Discovery (DAVID) integrated database query tools (http://david.abcc.ncifcrf.gov/) [25], [26]. A P-value was calculated to determine whether GO term or pathway annotates a specified list of genes at a frequency greater than that would be expected by chance. Bonferroni correction was adopted for multiple testing.

## Results

By searching the GWAS Integrator [15] and PheGenI (www.ncbi.nlm.nih.gov/gap/PheGenI/), we selected 135 robust genetic associations (P<10^−7^) between SLE and 128 unique SNPs (**Table S1**). These association results come from 8 published SLE association studies [6]–[13]. Some of these studies have relatively large sample sizes, e.g., the study performed by Han et.al. [6] has 1,047 Chinese Han cases and 1,205 Chinese Han controls plus 3,152 Chinese Han cases and 7,050 Chinese Han controls in initial and replication analyses, respectively. As shown in Table S1, the majority of these associations (>100 associations) were mapped to the HLA region. These SLE-associated genetic variants in HLA region span ∼6.4 M in length according to the physical location at chromosome 6p21.3.

As shown in **Table S1**, 7 SNPs have replication associations in multiple independent studies, e.g., rs7574865 in the intron region of STAT4 gene was significantly associated with SLE (P = 5.0E-42, and P = 2.0E-20) in two independent studies. The columns “Gene 1” and “Gene 2” listed the nearest genes physically located at two sides of the SNP, respectively. About 30 “susceptible” genes detected by GRAIL analysis, as listed in the column “implicated genes” of **Table S1**, are overlapped with the genes identified according to the physical positions (Columns “Gene 1” and “Gene 2”). The above two methods identified a total of 108 unique candidate genes, which were subject to further analyses.

EQTL analysis is an important approach in detection of functional mechanism underlying association by testing whether identified variants at the DNA level may lead to variations in mRNA expression of nearby genes. Among the 128 unique SNPs, we found that about 61 SNPs have potential eQTL effects either on the “identified” genes or on other genes (**Table 1** and **Table S2**). Fifty-five associations between 23 unique SNPs and expression of 11 “identified” genes from the list of **Table S1** (108 genes in total) were detected (**Table 1**) in monocytes and LCLs, which are two types of cells closely correlated with immune response. Among the 23 unique SNPs, only three were located at non-HLA regions. The entire 23 unique SNPs act as cis-effect regulators of the 11 “identified” genes. Especially, nine SNPs around HLA-DQB1 gene showed strong associations with expression level of HLA-DQB1 gene in two cell groups (LCLs and monocytes). Six SNPs (rs7192, rs9268832, rs9275224, rs2647012, rs9275572, and rs9275596), which extend more than 240 kb, were associated with the expression of HLA-DRA gene.

**Table 1:** Expression quantitative trait locus (eQTL) analysis results between SNPs and the expressions of 11 genes from the 108 “identified” genes.

SNP ID	Chr.	Location	Allele	Role	Association P-value	Gene 1	Gene 2	Implicated Gene	eQTL gene	Effect	Score	Target	Ref.
rs4728142	chr7	128573967	A/G	unknown	8.00E-19	KCP	IRF5	IRF5	IRF5	eqtl	130.5	Monocytes	[18]
										eqtl	8.6	LCLs	[20]
rs1573649	chr6	32731258	C/T	5′utr	2.00E-10	HLA-DQB2	HLA-DQB2	HLA-DQB2	HLA-DQB2	PP-eqtl	0.1	LCLs	[19]
rs6903130	chr6	32732210	A/G	upstream	4.43E-10	HLA-DQB2	HLA-DQB2	HLA-DQB2	HLA-DQB2	PP-eqtl	0	LCLs	[19]
rs16898264	chr6	32677152	A/G	unknown	3.85E-09	HLA-DQB1	HLA-DQA2	HLA-DRA	HLA-DQB1	exonQTL	4.4	LCLs	[22]
										transcriptQTL	4	LCLs	[22]
rs2647012	chr6	32664458	A/G	unknown	1.27E-13	HLA-DQB1	HLA-DQA2	HLA-DRA	HLA-DQB1	exonQTL	6.9	LCLs	[22]
										transcriptQTL	5.6	LCLs	[22]
rs2647050	chr6	32669767	C/T	unknown	5.38E-09	HLA-DQB1	HLA-DQA2	HLA-DRA	HLA-DQB1	exonQTL	4.4	LCLs	[22]
										transcriptQTL	4	LCLs	[22]
rs2856717	chr6	32670308	C/T	unknown	1.03E-13	HLA-DQB1	HLA-DQA2	N/A	HLA-DQB1	eqtl	33.9	Monocytes	[18]
										exonQTL	6.3	LCLs	[22]
										transcriptQTL	4.8	LCLs	[22]
rs2856718	chr6	32670255	A/G	unknown	5.33E-09	HLA-DQB1	HLA-DQA2	HLA-DRA	HLA-DQB1	exonQTL	4.4	LCLs	[22]
										transcriptQTL	4.1	LCLs	[22]
rs2856725	chr6	32666738	A/G	unknown	1.11E-13	HLA-DQB1	HLA-DQA2	N/A	HLA-DQB1	exonQTL	6.4	LCLs	[22]
										transcriptQTL	4.9	LCLs	[22]
rs2858305	chr6	32670464	A/C	unknown	7.88E-14	HLA-DQB1	HLA-DQA2	N/A	HLA-DQB1	exonQTL	6.8	LCLs	[22]
										transcriptQTL	5.3	LCLs	[22]
rs9275572	chr6	32678999	A/G	unknown	6.41E-14	HLA-DQB1	HLA-DQA2	HLA-DRA	HLA-DQB1	eqtl	24.3	Monocytes	[18]
										exonQTL	7.1	LCLs	[22]
										transcriptQTL	5.7	LCLs	[22]
rs9275596	chr6	32681631	C/T	unknown	3.33E-16	HLA-DQB1	HLA-DQA2	HLA-DRA	HLA-DQB1	exonQTL	5.3	LCLs	[22]
										transcriptQTL	4.2	LCLs	[22]
rs2187668	chr6	32605884	A/G	intron	6.00E-28	HLA-DQA1	HLA-DQA1	HLA-DQB2	HLA-DQA1	eqtl	34.6	Monocytes	[18]
										exonQTL	4.3	LCLs	[22]
rs9271100	chr6	32576478	C/T	unknown	1.00E-12	HLA-DRB1	HLA-DQA1	N/A	HLA-DQA1	exonQTL	7.8	LCLs	[22]
										transcriptQTL	5.4	LCLs	[22]
rs3094061	chr6	30321189	G/T	unknown	1.24E-09	HLA-N	UBQLN1P1	IER3	HLA-H	eqtl	33.6	Monocytes	[18]
rs9271100	chr6	32576478	C/T	unknown	1.00E-12	HLA-DRB1	HLA-DQA1	N/A	HLA-DRB1	exonQTL	15.2	LCLs	[22]
										transcriptQTL	10.5	LCLs	[22]
rs2647012	chr6	32664458	A/G	unknown	1.27E-13	HLA-DQB1	HLA-DQA2	HLA-DRA	HLA-DRA	transcriptQTL	4.5	LCLs	[22]
rs7192	chr6	32411646	G/T	missense	2.02E-10	HLA-DRA	HLA-DRA	HLA-DRA	HLA-DRA	transcriptQTL	9	LCLs	[22]
rs9268832	chr6	32427789	C/T	unknown	1.53E-10	HLA-DRB9	HLA-DRB9	HLA-DRA	HLA-DRA	transcriptQTL	9.5	LCLs	[22]
rs9275224	chr6	32659878	A/G	unknown	4.45E-12	HLA-DQB1	HLA-DQA2	HLA-DRA	HLA-DRA	transcriptQTL	4	LCLs	[22]
rs9275572	chr6	32678999	A/G	unknown	6.41E-14	HLA-DQB1	HLA-DQA2	HLA-DRA	HLA-DRA	eqtl	40.1	Monocytes	[18]
										transcriptQTL	5.1	LCLs	[22]
rs9275596	chr6	32681631	C/T	unknown	3.33E-16	HLA-DQB1	HLA-DQA2	HLA-DRA	HLA-DRA	transcriptQTL	4.7	LCLs	[22]
rs2647012	chr6	32664458	A/G	unknown	1.27E-13	HLA-DQB1	HLA-DQA2	HLA-DRA	HLA-DQA2	transcriptQTL	4.2	LCLs	[22]
rs2856725	chr6	32666738	A/G	unknown	1.11E-13	HLA-DQB1	HLA-DQA2	N/A	HLA-DQA2	transcriptQTL	4.2	LCLs	[22]
rs2858305	chr6	32670464	A/C	unknown	7.88E-14	HLA-DQB1	HLA-DQA2	N/A	HLA-DQA2	transcriptQTL	4.1	LCLs	[22]
rs9271100	chr6	32576478	C/T	unknown	1.00E-12	HLA-DRB1	HLA-DQA1	N/A	HLA-DRB1	eqtl	93	LCLs	Database#
rs13277113	chr8	11349186	A/G	unknown	1.00E-10	FAM167A	BLK	BLK	FAM167A	exonQTL	6.4	LCLs	[22]
										transcriptQTL	6.5	LCLs	[22]
rs12176317	chr6	26372786	A/G	intron	6.83E-10	BTN3A2	BTN3A2	TRIM38	BTN3A2	eqtl	21.4	LCLs	[19]
										exonQTL	4.8	LCLs	[22]
										PP-eqtl	0	LCLs	[19]
										transcriptQTL	10	LCLs	[22]
rs9379858	chr6	26367689	C/T	intron	6.21E-10	BTN3A2	BTN3A2	TRIM38	BTN3A2	eqtl	21.4	LCLs	20]
										PP-eqtl	0	LCLs	20]
rs9379859	chr6	26369549	C/T	intron	3.79E-10	BTN3A2	BTN3A2	TRIM38	BTN3A2	eqtl	21.4	LCLs	20]
										exonQTL	8.4	LCLs	[22]
										PP-eqtl	0	LCLs	20]
										transcriptQTL	10	LCLs	[22]
rs2618476	chr8	11352541	C/T	intron	2.00E-08	BLK	BLK	BLK	BLK	eqtl	57.1	LCLs	Database#

Note: N/A:not available; “Score”: −log10 (P); LCLs: lymphoblastoid cell lines; transcript-QTL: transcript expression levels against SNPs; exon-QTL: quantified reads for known exons by RNA sequencing against SNPs; sQTL: splicing QTL; PP-eqtl: posterior probability-eqtl.

All the eqtls have cis-effects.

Database#: http://eqtl.uchicago.edu/cgi-bin/gbrowse/eqtl.

“Gene 1” and “Gene 2”are the nearby genes physically located at two sides of SNP.

“GRAIL P-value “and “Implicated gene” are the results from Gene Relationships Across Implicated Loci (GRAIL) analyses.

Furthermore, we performed differential expression analyses for the 11 identified eQTL genes in multiple SLE related cells: macrophages, mononuclear cells, and peripheral blood cells (**Table 2**). T-tests showed that four genes (HLA-DQA1, HLA-DQB1, HLA-DQB2, and IRF5) were differentially expressed in samples S1 or S3 (**Table 2**).

**Table 2:** Differential expression analyses for the detected eQTL genes in multiple SLE related cells.

Sample	S1	S2	S3
**Disease**	SLE	SLE	SLE
**Target cells**	Macrophages	Mononuclear cells	Peripheral blood cells
**Sample size**	20∶16	20∶16	21∶45
**Platform**	Illumina HumanHT-12 V4.0 expression beadchip	Illumina HumanHT-12 V4.0 expression beadchip	Hitachisoft AceGene Human Oligo Chip 30K 1 Chip
**Ref.**	Database#	Database#	[14]
**GSE NO.**	GSE37356	GSE37356	GSE20864

Gene symbol	Probe ID	T-test P-value	SLE/control	Probe ID	T-test	SLE/control	Probe ID	T-test P-value	SLE/Control*
HLA-DQA1	ILMN_1808405	3.10E-01	1.04	ILMN_1808405	6.86E-01	1.01	AGhsA101413	4.88E-02	16.19048
HLA-DQB1	ILMN_1661266	6.99E-01	1.02	ILMN_1661266	9.83E-01	1	AGhsA030823	4.77E-02	6.2/5.08
HLA-DQB2	ILMN_1741648	2.49E-02	1.01	ILMN_1741648	6.25E-02	0.99	N/A	N/A	N/A
IRF5	ILMN_1670576	5.67E-01	0.98	ILMN_1670576	5.68E-01	0.98	AGhsB110506	1.62E-02	8.89/5.95

Note: Sample size: SLE cases: controls; N/A: Not available;

* :ratio of mean expression values of log2(sample/reference).

GSE NO: Gene Expression Omnibus Number, www.ncbi.nlm.nih.gov/geo/.

Database#: The data downloaded from the database (www.ncbi.nlm.nih.gov/geo/) according to GSE number.

We only listed the most significant expression results of probes if one gene has multiple detected probes.

The above functional evidence, taken together, highlighted the significance of 14 SLE-associated SNPs, i.e., the first 14 SNPs listed in Table 1, as these SNPs serve as cis-effect regulators of four SLE-associated genes (HLA-DQA1, HLA-DQB1, HLA-DQB2, and IRF5), which also were differentially expressed in SLE-related cell groups. Functional analysis using FASTSNP program [24] found that the rs2187668 in the intron of HLA-DQA1 was possible transcriptional binding sites for intronic enhancers. Therefore, the SNP may regulate transcription by altering binding sites for transcription factors or by increasing or decreasing the affinity of binding for transcription factors. Functions for the other 13 SNPs are unknown yet.

To test the probability of our “identified” genes clustering into a particular biological pathway and GO term, we performed a functional annotation clustering analysis using functional annotation tool of DAVID database. We found significant GO terms and KEGG pathways even after Bonferroni correction. The “identified” genes tend to enrich in immune-related pathways (such as “autoimmune thyroid disease”, “Intestinal immune network for IgA production”, and “immune response”) (data not shown). Especially, we found a significant clustering (Bonferroni correction P = 8.00E-06) of 8 genes (HLA-DQB1, TNF, HLA-DRB1, HIST1H2AH, HLA-DOB, HLA-DQA2, HLA-DQA1, HLA-DRA) directly involved in SLE. Among the 11 identified eQTL genes, five genes (HLA-DQB1, HLA-DRB1, HLA-DQA2, HLA-DQA1, and HLA-DRA) were significantly clustered in multiple immune related KEGG pathways or GO terms (**Table S3**).

## Discussion

By using publically available datasets, this study performed integrative analyses and combined with eQTL results to detect functional mechanisms underlying the associations for SLE. We discovered 23 SNPs acting as cis-effect regulators on 11 “identified” genes. Four eQTL genes have differential expression signals in the SLE-associated cells or tissues. The “identified” genes tend to enrich in KEGG Pathways (autoimmune thyroid disease, intestinal immune network for IgA production, and immune response), which are very closely relevant to SLE.

In biologic systems, genetic information carried by DNA is passed on to RNA molecules via transcription and then to protein molecules through translation. Sequence variants at the DNA level represent a class of heritable molecules and contribute to variability of complex traits in the population. The functional mechanisms underlying associations between variants at the DNA level and SLE may be that DNA sequence variants lead to variation in quantifiable intermediate phenotypes (such as RNA level), which subsequently lead to variation of susceptibility to SLE. Therefore, integrating substantial evidences from multiple levels (i.e., DNA, RNA) could ascertain potential functioning mechanism of genes and their contribution to variation in susceptibility to SLE.

Recent association studies have identified a long list of loci that were associated with SLE with relatively high statistical power. A majority of these associations (>100 associations) were mapped to the HLA region. It is well-known that there is strong linkage disequilibrium (LD) at HLA region. It is reasonable to infer that the significant signals for some of the genetic markers may be due to their strong LD with true functional variants within the HLA region. As shown in Results section, multiple SNPs, extending a relatively long distance (240 kb), were consistently associated with the expression of HLA-DRA. Strong LD at HLA region may partially contribute to this observation.

HLA-DQA1, HLA-DQB1, and HLA-DQB2 are HLA class II genes involved in immune response. Class II molecules are heterodimers consisting of an alpha (DQA) and a beta chain (DQB), both anchored to the membrane, which play a central role in the immune system by presenting peptides derived from extracellular proteins. These genes have been found to consistently associate with SLE in several populations [7], [8] at DNA level, though functions for these genes and mechanism for the observed associations were not studied. The functional evidence from this study, taken together, suggested potential regulatory mechanisms underlying the associations between SLE and the associated SNPs. Briefly, different genotypes of the SNPs, by regulating differential mRNA transcriptions of functional genes (HLA-DQA1, HLA-DQB1, and HLA-DQB2) and thus differential protein expression and enzyme activity, consequently effect on variation of susceptibility to SLE in the population.

Interferon (IFN) regulatory factor 5 (IRF5) is a pivotal transcription factor in the type I IFN pathway, which regulates inflammatory cytokines, expression of IFN-dependent genes, and genes involved in apoptosis. Previous studies identified IRF5 as one of the most strongly and consistently SLE-associated loci outside the HLA region. Four functional variants in IRF5 were identified in multiple ethnic groups: a 5 bp indel (insertion–deletion) near the 5′ untranslated region (UTR), rs2004640 in the first intron, a 30 bp indel in the sixth exon, and rs10954213 in the 3′ UTR [27]–[35] . In addition, a SNP, rs4728142, located 10 kb downstream of IRF5 gene, was highly strongly associated with SLE (P = 8.00E-19) [6]. In the eQTL analysis, this SNP was consistently and highly associated with expression of IRF5 gene in both monocytes (P<E-130) [18] and lymphoblastoid cell lines (P<E-08) [30]. Compared with GG genotype at rs4728142, AA genotype individuals tend to have higher IRF5 expression levels. The combined evidence from association analyses, eQTL analyses, and differential expression analyses suggested that the rs4728142 or other nearby genetic variant contribute significant to variation of susceptibility to SLE in the population.

To be noted, failure to find functional evidence underlying the selected associations through mining publically available data and results does not necessarily exclude the importance of the associations with SLE, given that (1) absence of association between SLE-associated SNP and mRNA expression may result from limited statistical power due to small sample size or small effect of the SNP; (2) the associated genes may exert effects on SLE through other tissues or cells not studied herein; (3) sequence variant in a gene may lead to SLE by other mechanisms (e.g., epigenetic regulation) instead of regulating gene expression. In-depth functional studies are required, which may provide novel insights into mechanisms underlying the associations detected at DNA level.

In addition, this exploratory pilot study has two potential limitations. First, although we gained some functional evidence for the associations between SLE and 14 SNPs, based on the current available evidence, it is very challenging to discriminate causal variants with nearby SNPs with potential functional effects, especially for SNPs at the HLA region where there is strong LD. Second, although we used two methods to select the potential genes involved in associations (gene selection according to the physical location and GRAIL analysis based on previous literature), this study does not exclude the possibility that other genes around the SNPs are also directly involved in the association around SNPs.

In summary, the functional evidence gained in this study, taken together, supports and highlights the significance of 14 SLE-associated SNPs. These SNPs act as cis-effect regulators to regulate expression of genes that were found differentially expressed between SLE patients and controls. The above evidence laid a supporting clue for us to pursue in-depth validation studies to dissect their involvements and molecular functional mechanisms in SLE. This study sets an example of mining publically available datasets and results to validate significant disease-association results. Utilization of public data resources for integrative analyses may provide novel insights into the molecular genetic mechanisms underlying human diseases.

## Supporting Information

Table S1 The selected association results (p<10-7) from previous 8 published studies. Note: N/A: not available; PMID: PubMed ID. “Gene 1” and “Gene 2” are the nearby genes physically located at two sides of SNP. “GRAIL p-value” and “Implicated gene” are the results from Gene Relationships Across Implicated Loci (GRAIL) analyses. * associated with anti-dsDNA negative SLE; #associated with anti-dsDNA positive SLE.
(XLS)

Table S2 Results of eQTL analyses for the selected significant SLE-associated SNPs that have eQTL effects not on the corresponding “identified” genes but on other genes. Note: N/A:not available;“Score”: −log10 (P); LCLs: lymphoblastoid cell lines; transcript-QTL: transcript expression levels against SNPs; exon-QTL: quantified reads for known exons by RNA sequencing against SNPs; sQTL: splicing QTL; PP-eqtl: posterior probability-eqtl; Database#: http://eqtl.uchicago.edu/cgi-bin/gbrowse/eqtl; PMID for eQTL: PubMed ID for eQTL results. “Gene 1” and “Gene 2”are the nearby genes physically located at two sides of SNP. “GRAIL p-value “and “Implicated gene” are the results from Gene Relationships Across Implicated Loci (GRAIL) analyses.
(XLS)

Table S3 Functional annotation clustering analysis for the eQTL genes. Note: Functional annotation clustering analysis was performed using functional annotation tool of DAVID database.
(DOCX)
