# Supplementary material for: Functional Relevance for Associations between Genetic Variants and Systemic Lupus Erythematosus
Source: PLoS One. 2013 Jan 14;8(1):e53037. doi: 10.1371/journal.pone.0053037 (PMC3544818; doi:10.1371/journal.pone.0053037)
Supplement: Table S3 — Functional annotation clustering analysis for the eQTL genes. Note: Functional annotation clustering analysis was performed using functional annotation tool of DAVID database. (DOCX) [file pone.0053037.s003.docx]

**Table S3. Functional annotation clustering analysis for the eQTL genes**

| **Category** | **Term** | **Count** | **%** | **P -value** | **Genes** | **List Total** | **Pop**  **Hits** | **Pop Total** | **Fold Enrichment** | **Bonferroni** |
| --- | --- | --- | --- | --- | --- | --- | --- | --- | --- | --- |
| KEGG_PATHWAY | hsa05310:Asthma | 5 | 45.45 | 4.25E-09 | HLA-DQB1, HLA-DRB1, HLA-DQA2, HLA-DQA1, HLA-DRA | 6 | 29 | 5085 | 146.12 | 5.10E-08 |
| KEGG_PATHWAY | hsa05330:Allograft rejection | 5 | 45.45 | 1.05E-08 | HLA-DQB1, HLA-DRB1, HLA-DQA2, HLA-DQA1, HLA-DRA | 6 | 36 | 5085 | 117.71 | 1.26E-07 |
| KEGG_PATHWAY | hsa05332:Graft-versus-host disease | 5 | 45.45 | 1.47E-08 | HLA-DQB1, HLA-DRB1, HLA-DQA2, HLA-DQA1, HLA-DRA | 6 | 39 | 5085 | 108.65 | 1.76E-07 |
| KEGG_PATHWAY | hsa04940:Type I diabetes mellitus | 5 | 45.45 | 2.00E-08 | HLA-DQB1, HLA-DRB1, HLA-DQA2, HLA-DQA1, HLA-DRA | 6 | 42 | 5085 | 100.89 | 2.40E-07 |
| KEGG_PATHWAY | hsa04672:Intestinal immune network for IgA production | 5 | 45.45 | 3.78E-08 | HLA-DQB1, HLA-DRB1, HLA-DQA2, HLA-DQA1, HLA-DRA | 6 | 49 | 5085 | 86.48 | 4.54E-07 |
| KEGG_PATHWAY | hsa05320:Autoimmune thyroid disease | 5 | 45.45 | 4.46E-08 | HLA-DQB1, HLA-DRB1, HLA-DQA2, HLA-DQA1, HLA-DRA | 6 | 51 | 5085 | 83.09 | 5.35E-07 |
| KEGG_PATHWAY | hsa05416:Viral myocarditis | 5 | 45.45 | 1.73E-07 | HLA-DQB1, HLA-DRB1, HLA-DQA2, HLA-DQA1, HLA-DRA | 6 | 71 | 5085 | 59.68 | 2.07E-06 |
| KEGG_PATHWAY | hsa04612:Antigen processing and presentation | 5 | 45.45 | 3.26E-07 | HLA-DQB1, HLA-DRB1, HLA-DQA2, HLA-DQA1, HLA-DRA | 6 | 83 | 5085 | 51.05 | 3.91E-06 |
| KEGG_PATHWAY | hsa05322:Systemic lupus erythematosus | 5 | 45.45 | 6.66E-07 | HLA-DQB1, HLA-DRB1, HLA-DQA2, HLA-DQA1, HLA-DRA | 6 | 99 | 5085 | 42.80 | 8.00E-06 |
| KEGG_PATHWAY | hsa04514:Cell adhesion molecules (CAMs) | 5 | 45.45 | 2.13E-06 | HLA-DQB1, HLA-DRB1, HLA-DQA2, HLA-DQA1, HLA-DRA | 6 | 132 | 5085 | 32.10 | 2.55E-05 |
| GOTERM_BP_FAT | GO:0019882~antigen processing and presentation | 6 | 54.55 | 1.60E-10 | HLA-DQB1, HLA-H, HLA-DRB1, HLA-DQA2, HLA-DQA1, HLA-DRA | 8 | 83 | 13528 | 122.24 | 4.96E-09 |
| GOTERM_BP_FAT | GO:0002504~antigen processing and presentation of peptide or polysaccharide antigen via MHC class II | 5 | 45.45 | 1.02E-09 | HLA-DQB1, HLA-DRB1, HLA-DQA2, HLA-DQA1, HLA-DRA | 8 | 33 | 13528 | 256.21 | 3.17E-08 |
| GOTERM_BP_FAT | GO:0006955~immune response | 6 | 54.55 | 6.56E-06 | HLA-DQB1, HLA-H, HLA-DRB1, HLA-DQA2, HLA-DQA1, HLA-DRA | 8 | 690 | 13528 | 14.70 | 2.03E-04 |
| Note: Functional annotation clustering analysis was performed using functional annotation tool of DAVID database. | | | | | | | | | | |
